# Supplementary material for: Three-dimensional chromatin reorganization regulates B cell development during ageing
Source: Nat Cell Biol. 2024 Jun 12;26(6):991–1002. doi: 10.1038/s41556-024-01424-9 (PMC11178499; doi:10.1038/s41556-024-01424-9)
Supplement: Supplementary file 1 — Supplementary Fig. 1. [file 41556_2024_1424_MOESM1_ESM.pdf]

nature cell biology

Supplementary information https://doi.org/10.1038/s41556-024-01424-9

Three-dimensional chromatin reorganization regulates B cell development during ageing

In the format provided by the authors and unedited

Gating stragety of Extended Figure 1a.

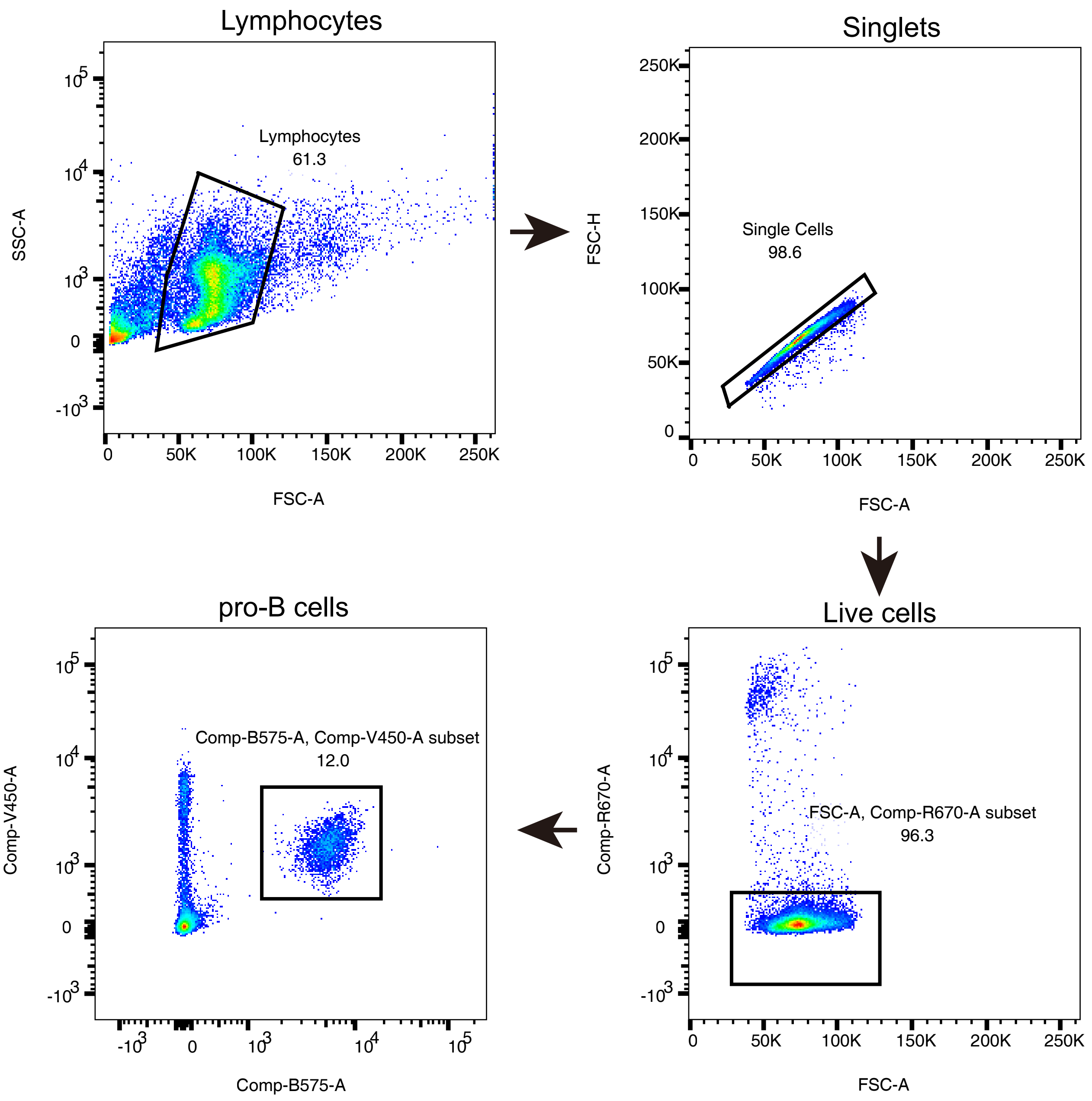

One young *Rag2*$^{-/-}$ mouse shown as the example.
