# Supplementary material for: Three-dimensional chromatin reorganization regulates B cell development during ageing
Source: Nat Cell Biol. 2024 Jun 12;26(6):991–1002. doi: 10.1038/s41556-024-01424-9 (PMC11178499; doi:10.1038/s41556-024-01424-9)
Supplement: Supplementary file 2 — Reporting Summary [file 41556_2024_1424_MOESM2_ESM.pdf]

nature portfolio

Corresponding author(s): Ranjan Sen

Last updated by author(s): Mar 26, 2024

Reporting Summary

Nature Portfolio wishes to improve the reproducibility of the work that we publish. This form provides structure for consistency and transparency in reporting. For further information on Nature Portfolio policies, see our Editorial Policies and the Editorial Policy Checklist.

Statistics

For all statistical analyses, confirm that the following items are present in the figure legend, table legend, main text, or Methods section.

n/a	Confirmed	
☐	☒	The exact sample size (*n*) for each experimental group/condition, given as a discrete number and unit of measurement
☐	☒	A statement on whether measurements were taken from distinct samples or whether the same sample was measured repeatedly
☐	☒	The statistical test(s) used AND whether they are one- or two-sided<br>*Only common tests should be described solely by name; describe more complex techniques in the Methods section.*
☐	☒	A description of all covariates tested
☐	☒	A description of any assumptions or corrections, such as tests of normality and adjustment for multiple comparisons
☐	☒	A full description of the statistical parameters including central tendency (e.g. means) or other basic estimates (e.g. regression coefficient) AND variation (e.g. standard deviation) or associated estimates of uncertainty (e.g. confidence intervals)
☐	☒	For null hypothesis testing, the test statistic (e.g. *F*, *t*, *r*) with confidence intervals, effect sizes, degrees of freedom and *P* value noted<br>*Give P values as exact values whenever suitable.*
☒	☐	For Bayesian analysis, information on the choice of priors and Markov chain Monte Carlo settings
☒	☐	For hierarchical and complex designs, identification of the appropriate level for tests and full reporting of outcomes
☐	☒	Estimates of effect sizes (e.g. Cohen's *d*, Pearson's *r*), indicating how they were calculated

Our web collection on statistics for biologists contains articles on many of the points above.

Software and code

Policy information about availability of computer code

Data collection	No softwares were used to collect public data. Sequencing data in FASTQ format were transferred by sequencing core with Google Drive or Globus (v3.1.5.637).
Data analysis	cLoops2 (v0.0.2): https://github.com/YaqiangCao/cLoops2<br>Bowtie2 (v2.3.5): http://bowtie-bio.sourceforge.net/bowtie2/index.shtml<br>STAR (v2.7.3a): https://github.com/alexdobin/STAR<br>Cufflinks (v2.2.1): http://cole-trapnell-lab.github.io/cufflinks/<br>HiCUP (v0.7.2): https://www.bioinformatics.babraham.ac.uk/projects/hicup<br>HiCExplorer3 (v3.6): https://hicexplorer.readthedocs.io/en/latest/<br>Juiicer (v1.11.04): https://github.com/aidenlab/juicer<br>deepTools (v3.3.0): https://deeptools.readthedocs.io/en/develop/index.html<br>Flowjo (10.6.0):https://www.flowjo.com/solutions/flowjo/downloads/previous-versions<br>GraphPad Prism (v.9.5.0):https://www.graphpad.com/updates/prism-900-release-notes

For manuscripts utilizing custom algorithms or software that are central to the research but not yet described in published literature, software must be made available to editors and reviewers. We strongly encourage code deposition in a community repository (e.g. GitHub). See the Nature Portfolio guidelines for submitting code & software for further information.

Data

Policy information about availability of data

All manuscripts must include a data availability statement. This statement should provide the following information, where applicable:

- Accession codes, unique identifiers, or web links for publicly available datasets
- A description of any restrictions on data availability
- For clinical datasets or third party data, please ensure that the statement adheres to our policy

Hi-C, Capture Hi-C, RNA-Seq, ChIP-seq, HiChIP and VDJ-Seq data generated by this study have been deposited to GEO with public accession of GSE214438. Both raw data and processed data with mouse reference genome mm10 are provided.

Human research participants

Policy information about studies involving human research participants and Sex and Gender in Research.

Reporting on sex and gender	N/A
Population characteristics	N/A
Recruitment	N/A
Ethics oversight	N/A

Note that full information on the approval of the study protocol must also be provided in the manuscript.

Field-specific reporting

Please select the one below that is the best fit for your research. If you are not sure, read the appropriate sections before making your selection.

☒ Life sciences ☐ Behavioural & social sciences ☐ Ecological, evolutionary & environmental sciences

For a reference copy of the document with all sections, see nature.com/documents/nr-reporting-summary-flat.pdf

Life sciences study design

All studies must disclose on these points even when the disclosure is negative.

Sample size	No statistical method was used to pre-determine the sample size. The sample sizes used in this study are indicated in the corresponding legends. Our sample sizes are similar to those reported in previous publications about aged mice.
Data exclusions	No data were excluded for analysis.
Replication	Biological replicates of each experiments were described in the corresponding figure legends.
Randomization	No randomization was needed for data collection. Some presentation examples were randomly selected.
Blinding	The sequencing data in this study were processed and analyzed with computer programs. The investigators were not blinded during data collection and analysis. No blinding was required in this study.

Reporting for specific materials, systems and methods

We require information from authors about some types of materials, experimental systems and methods used in many studies. Here, indicate whether each material, system or method listed is relevant to your study. If you are not sure if a list item applies to your research, read the appropriate section before selecting a response.

Materials & experimental systems

n/a	Involved in the study
☐	☒ Antibodies
☐	☒ Eukaryotic cell lines
☒	☐ Palaeontology and archaeology
☐	☒ Animals and other organisms
☒	☐ Clinical data
☒	☐ Dual use research of concern

Methods

n/a	Involved in the study
☐	☒ ChIP-seq
☐	☒ Flow cytometry
☒	☐ MRI-based neuroimaging

Antibodies

Antibodies used

PE anti-CD19 (BioLegend, 6D5, Cat#115508)
BV421 anti-B220 (BioLegend, RA3-6B2, Cat # 103240)
FITC anti-IgM (BioLegend, RMM-1, Cat # 4065060)
PE anti-CD43 (BD Biosciences, S7, Cat #553271)
H3K27ac-AF488 conjugated (Cell Signaling Technology, Cat #15485S)
IgG isotype control-AF488 conjugated (Cell Signaling Technology, Cat #4340S)
anti-H3K27ac (Active Motif, Cat # 39133),
anti-H3K27me3 (Diagonode, Cat #C1541005)
anti-CTCF (Abcam, Cat# ab703030)
anti-Rad21 (Abcam, Cat# ab992)
anti-Brg1 (Abcam, Cat #ab110641)
anti-p300 (Cell Signaling Technology, Cat#57625S)

Validation

The validation information can be found in the merchandise websites. Each antibody was titrated before use and the dilution ratio is shown below:
Flow cytometry:
PE anti-CD19 (BioLegend, 6D5, Cat#115508, 1:100. Verified Reactivity: Mouse. Application: FC. https://www.biolegend.com/ja-jp/products/pe-anti-mouse-cd19-antibody-1530)
BV421 anti-B220 (BioLegend, RA3-6B2, Cat # 103240, 1:100. Verified Reactivity:Mouse, Human. Application: FC/IHC-F. https://www.biolegend.com/nl-be/products/brilliant-violet-421-anti-mouse-human-cd45r-b220-antibody-7158)
FITC anti-IgM (BioLegend, RMM-1, Cat # 406506; 1:50. Verified Reactivity:Mouse. Application: FC. https://www.biolegend.com/de-de/products/fitc-anti-mouse-igm-2334?GroupID=BLG3548)
PE anti-CD43 (BD Biosciences, S7, Cat #553271, 1:100. Verified Reactivity: Mouse. Application: FC. https://www.bdbiosciences.com/en-us/products/reagents/flow-cytometry-reagents/research-reagents/single-color-antibodies-ruo/pe-rat-anti-mouse-cd43.553271)
H3K27ac-AF488 conjugated (Cell Signaling Technology, Cat #15485S. 1:50. Verified Reactivity:Mouse, Human, Rat, Monkey. Application: FC/IHC-F. https://www.cellsignal.com/products/antibody-conjugates/acetyl-histone-h3-lys27-d5e4-xp-rabbit-mab-alexa-fluor-488-conjugate/15485)
IgG isotype control-AF488 conjugated (Cell Signaling Technology, Cat #4340S; 1:50. Verified Reactivity:Mouse, Human. Application: FC. https://www.cellsignal.com/products/antibody-conjugates/rabbit-igg-isotype-control-alexa-fluor-488-conjugate/4340)
ChIP-Seq and Hi-ChIP:
anti-H3K27ac (Active Motif, Cat # 39133; 1:500. Verified Reactivity:Mouse, Human, Budding Yeast. Application: FC/IHC-F/IP/ChIP/WB/ICC/CUT&RUN/CUT&Tag. https://www.activemotif.com/catalog/details/39133/histone-h3-acetyl-lys27-antibody-pab)
anti-H3K27me3 (Diagonode, Cat #C15410195; 1:500. Verified Reactivity:Mouse, Human, Drosophila, C. elegans, Daphnia, Arabidopsis, maize, tomato, poplar, silena latifolia, C. merolae, wide range expected. Application: WB/IHC-Fr/IP/CUT&Tag/ELISA/ChIP. https://www.diagenode.com/en/p/h3k27me3-polyclonal-antibody-premium-50-mg-27-ml)
anti-CTCF (Abcam, Cat# ab70303; 1:250. Verified Reactivity:Mouse, Human. Application: WB/IHC-Fr/IP/IHC-P. https://www.abcam.com/products/primary-antibodies/ctcf-antibody-ab70303.html)
anti-Rad21 (Abcam, Cat# ab992; 1:500. Verified Reactivity:Mouse, Human. Application: WB/IP. https://www.abcam.com/products/primary-antibodies/rad21-antibody-ab992.html)
anti-Brg1 (Abcam, Cat #ab110641; 1:200. Mouse, Human, Rad. Application: FC/IHC-P/IP/ICC/WB/IF/CUT&RUN. https://www.abcam.com/products/primary-antibodies/brg1-antibody-epncir111a-ab110641.html)
anti-p300 (Cell Signaling Technology, Cat#57625S; 1:333. Verified Reactivity:Mouse, Human, Rat, Monkey. Application: WB/IP/ChIP/CUT&RUN)

Eukaryotic cell lines

Policy information about cell lines and Sex and Gender in Research

Cell line source(s)

The D345 cell line was generously provided by David G. Schatz of Yale University.
293T human embryonic kidney cells used for lentiviral expression was purchased from ATCC (Cat# CRL-3216).
The Platinum-E (Plat-E) cell line used for retroviral expression was purchased from Cell Biolabs (Cat# RV-101).
Rag2-/-Ebf1+/+Pax5+/+, Rag2-/-Ebf1+/-Pax5+/+ and Rag2-/-Ebf1+/–Pax5+/– pro-B cells were provided by Rudolf Grosschedl of Max Planck Institute of Immunobiology and Epigenetics.

Authentication

Cell lines were authenticated by morphology and genotyping.

Mycoplasma contamination

We did not test the mycoplasma contamination for these cells.

Commonly misidentified lines (See ICLAC register)	No commonly misidentified cell lines were used in this study.

Animals and other research organisms

Policy information about studies involving animals; ARRIVE guidelines recommended for reporting animal research, and Sex and Gender in Research

Laboratory animals	Young (2-3 months) recombination activating gene 2-deficent (Rag2-/-) mice were purchased from Jackson Lab (Stock# 008449) and maintained to be old (23-24 months) in house by NIA Comparative Medicine Section (CMS). Young (2-3 months) and old (23-24 months) C57BL/6J mice were provided by National Institute on Aging (NIA) Aged Rodent Colonies.
Wild animals	No wild animals were involved in this study.
Reporting on sex	This study did not specifically focus on sex differences, and for each experiment, the sexes of both young and old mice were matched.
Field-collected samples	No field-collected samples were involved in this study.
Ethics oversight	All mouse experiments were performed under protocols approved by NIA Institutional Animal Care and Use Committees (338-LMBI-2022,2023,2024,2025)

Note that full information on the approval of the study protocol must also be provided in the manuscript.

ChIP-seq

Data deposition

☒ Confirm that both raw and final processed data have been deposited in a public database such as GEO.

☒ Confirm that you have deposited or provided access to graph files (e.g. BED files) for the called peaks.

Data access links *May remain private before publication.*	ChIP-seq data generated by this study have been deposited to GEO with public accession of GSE214438
Files in database submission	Processed uniquely mapped high quality reads in BED or BEDPE files were uploaded to GEO for downloading and downstream analysis. Raw FASTQ files are available corresponding to the GSM accession.<br>GSM6605809_ChIP-seq_Old_CTCF_rep1.bed.gz<br>GSM6605810_ChIP-seq_Old_CTCF_rep2.bed.gz<br>GSM6605811_ChIP-seq_Old_H3K27ac_rep1.bed.gz<br>GSM6605812_ChIP-seq_Old_H3K27ac_rep2.bed.gz<br>GSM6605813_ChIP-seq_Old_H3K27me3_rep1.bed.gz<br>GSM6605814_ChIP-seq_Old_H3K27me3_rep2.bed.gz<br>GSM6605815_ChIP-seq_Old_Rad21_rep1.bed.gz<br>GSM6605816_ChIP-seq_Old_Rad21_rep2.bed.gz<br>GSM6605817_ChIP-seq_Young_CTCF_rep1.bed.gz<br>GSM6605818_ChIP-seq_Young_CTCF_rep2.bed.gz<br>GSM6605819_ChIP-seq_Young_H3K27ac_rep1.bed.gz<br>GSM6605820_ChIP-seq_Young_H3K27ac_rep2.bed.gz<br>GSM6605821_ChIP-seq_Young_H3K27me3_rep1.bed.gz<br>GSM6605822_ChIP-seq_Young_H3K27me3_rep2.bed.gz<br>GSM6605823_ChIP-seq_Young_Rad21_rep1.bed.gz<br>GSM6605824_ChIP-seq_Young_Rad21_rep2.bed.gz<br>GSM7921744 ChIP-seq_Old_Brg1_rep1.bed.gz<br>GSM7921745 ChIP-seq_Old_Brg1_rep2.bed.gz<br>GSM7921746 ChIP-seq_Old_p300_rep1.bed.gz<br>GSM7921747 ChIP-seq_Old_p300_rep2.bed.gz<br>GSM7921748 ChIP-seq_Young_Brg1_rep1.bed.gz<br>GSM7921749 ChIP-seq_Young_Brg1_rep2.bed.gz<br>GSM7921750 ChIP-seq_Young_p300_rep1.bed.gz<br>GSM7921751 ChIP-seq_Young_p300_rep2.bed.gz
Genome browser session (e.g. UCSC)	WashU Epigenome Browser (http://epigenomegateway.wustl.edu/browser/) session bundle id: cdc07070-8e21-11ee-946c-99128820ea7d

Methodology

Replicates	ChIP-seq in this study were performed with two biological replicates.

Sequencing depth

Single-end sequenced ChIP-seq samples:
sample TotalReads MappingRatio(%s) redundancy totalMappedReads uniqueReads
ChIP-seq_Old_CTCF_rep1 36152000 99.72 0.174670618 36003382 29714649
ChIP-seq_Old_CTCF_rep2 38403295 99.66 0.310768488 38219155 26341846
ChIP-seq_Old_H3K27ac_rep1 52414407 99.47 0.137581775 52051887 44890496
ChIP-seq_Old_H3K27ac_rep2 68395723 99.65 0.1378693 68048340 58666563
ChIP-seq_Old_H3K27me3_rep1 49286106 99.63 0.123891891 49012562 42940303
ChIP-seq_Old_H3K27me3_rep2 56635461 99.69 0.116566709 56357935 49788476
ChIP-seq_Old_Rad21_rep1 37594299 99.72 0.167368901 37439566 31173347
ChIP-seq_Old_Rad21_rep2 32376536 99.76 0.172744361 32258888 26686347
ChIP-seq_Young_CTCF_rep1 35139722 99.28 0.172994046 34843176 28815514
ChIP-seq_Young_CTCF_rep2 35386844 99.68 0.285618516 35224558 25163772
ChIP-seq_Young_H3K27ac_rep1 40703245 99.71 0.146787088 40534260 34584354
ChIP-seq_Young_H3K27ac_rep2 42092146 99.73 0.147615562 41922687 35734246
ChIP-seq_Young_H3K27me3_rep1 42840595 99.58 0.123732445 42591044 37321150
ChIP-seq_Young_H3K27me3_rep2 48840342 99.58 0.125633268 48555499 42455313
ChIP-seq_Young_Rad21_rep1 32632147 99.58 0.172958351 32461179 26846747
ChIP-seq_Young_Rad21_rep2 44822618 99.69 0.179776658 44629765 36606375

Paired-end sequenced ChIP-seq samples:
sample TotalReads MappingRatio(%s) redundancy totalMappedPETs uniquePETs
ChIP-seq_Old_Brg1_rep1 61783501 96.12 0.14960201 52364049 44530282
ChIP-seq_Old_Brg1_rep2 69004309 95.68 0.171523419 58220907 48234658
ChIP-seq_Old_p300_rep1 73935833 96.56 0.184713159 62098429 50628032
ChIP-seq_Old_p300_rep2 68687702 96.76 0.185678715 57910402 47157673
ChIP-seq_Young_Brg1_rep1 63872867 97.2 0.380600285 54827857 33960359
ChIP-seq_Young_Brg1_rep2 61142512 96.15 0.432857312 52074673 29533770
ChIP-seq_Young_p300_rep1 64604824 96.49 0.501838071 53618451 26710671
ChIP-seq_Young_p300_rep2 60880153 96.91 0.29125307 51002343 36147754

Antibodies

anti-H3K27ac (Active Motif, Cat # 39133; 1:500)
anti-H3K27me3 (Diagonode, Cat #C1541005; 1:500)
anti-CTCF (Abcam, Cat# ab70303; 1:250)
anti-Rad21 (Abcam, Cat# ab992; 1:500)
anti-Brg1 (Abcam, Cat #ab110641; 1:200)
anti-p300 (Cell Signaling Technology, Cat#57625S; 1:333)

Peak calling parameters

ChIP-seq raw reads were mapped to the mouse reference genome mm10 by Bowtie2 (v2.3.5). Only non-redundant reads with MAPQ >=10 were used for the following analysis. BigWig tracks were generated by bamCoverage in deepTools (v3.3.0) with parameters of --ignoreDuplicates --minMappingQuality 10 --normalizeUsing CPM for visualization and quantification aggregation analysis. Peaks were called by cLoops2 with key parameters of -eps 150 for CTCF and Rad21, -eps 150,300 for H3K27ac and -eps 300,500 for H3K27me3, and -minPts was set to 20,50.

Data quality

CTCF motifs were checked for CTCF and RAD21 ChIP-seq data. Visualization through genome browser also confirmed strong peaks of all samples. We also measured the ratio of peaks (show as following) that have higher than 5-folds signal enrichment compared to the mean value of upstream and downstream flanking regions of 5-fold, 10-fold and 20-fold sizes.

5-fold ES 10-fold ES 20-fold ES
ChIP-seq_Old_CTCF_rep1 88.68563422 91.1182034 98.62997704
ChIP-seq_Old_CTCF_rep2 84.22470872 85.45398553 99.30708823
ChIP-seq_Old_H3K27ac_rep1 43.99613099 47.81677491 67.65925107
ChIP-seq_Old_H3K27ac_rep2 35.20046564 37.76834321 58.78042935
ChIP-seq_Old_H3K27me3_rep1 17.69499418 22.99185099 34.90104773
ChIP-seq_Old_H3K27me3_rep2 15.6460945 19.75650916 29.74927676
ChIP-seq_Old_Rad21_rep1 79.41933113 82.10951856 94.63800074
ChIP-seq_Old_Rad21_rep2 84.13290535 86.55764726 98.6900321
ChIP-seq_Young_CTCF_rep1 89.63096983 91.25931934 98.96434476
ChIP-seq_Young_CTCF_rep2 87.59759687 89.30508598 99.61082869
ChIP-seq_Young_H3K27ac_rep1 57.53865751 60.89664202 77.03549061
ChIP-seq_Young_H3K27ac_rep2 57.23651918 60.08344436 75.92384826
ChIP-seq_Young_H3K27me3_rep1 25.87425595 30.74776786 43.34077381
ChIP-seq_Young_H3K27me3_rep2 26.41387582 30.27954608 40.62182858
ChIP-seq_Young_Rad21_rep1 85.96245307 87.34918648 99.1864831
ChIP-seq_Young_Rad21_rep2 79.14553486 80.6296305 91.30577044
ChIP-seq_Old_Brg1_rep1 16.21013424 23.16287732 30.34841313
ChIP-seq_Old_Brg1_rep2 19.78908583 26.4595642 33.73356204
ChIP-seq_Old_p300_rep1 43.5253268 45.93545752 36.90767974
ChIP-seq_Old_p300_rep2 43.48846304 46.71490027 36.8596011
ChIP-seq_Young_Brg1_rep1 32.89585819 44.02590762 56.84336117
ChIP-seq_Young_Brg1_rep2 49.37111265 62.44644091 86.49619903
ChIP-seq_Young_p300_rep1 65.48011426 71.02834542 78.78488244
ChIP-seq_Young_p300_rep2 74.39927733 79.90966576 91.36404697

Software	Bowtie2 (v2.3.5): http://bowtie-bio.sourceforge.net/bowtie2/index.shtml<br>deepTools (v3.3.0): https://deeptools.readthedocs.io/en/develop/index.html<br>cLoops2 (v0.0.2): https://github.com/YaqiangCao/cLoops2

Flow Cytometry

Plots

Confirm that:

☒ The axis labels state the marker and fluorochrome used (e.g. CD4-FITC).

☒ The axis scales are clearly visible. Include numbers along axes only for bottom left plot of group (a 'group' is an analysis of identical markers).

☒ All plots are contour plots with outliers or pseudocolor plots.

☒ A numerical value for number of cells or percentage (with statistics) is provided.

Methodology

Sample preparation	For Rag2-/- mice, total bone marrow was extracted from tibia and femurs, and erythrocytes are lysed. Pro-B cells were purified by combining positive selection using CD19+ selective beads (Stem Cell Technology, Cat#18954) and sorting by CD19 + and B220+ markers. For C57BL/6J mice, total bone marrow was extracted from tibia and femurs and erythrocytes are lysed. Cells were pre-purified using CD19+ selective beads (Stem Cell Technology, Cat#18954) and sorted with IgM-B220+CD43+ markers.
Instrument	BD FACSAria II
Software	BD FACSDiva and Flowjo_v10.6.0 software were used for data acquisition and analysis.
Cell population abundance	Cell purity was assessed after sorting, with more than 95% purity achieved after each sorting, ensuring suitability for subsequent experiments.
Gating strategy	For the Rag2-/- mice, cells were gated on FSC-A/SSC-A to select lymphocytes, FSC-H/FSC-A to gate on single cells. Then CD19 +B220+ cells were sorted for experiment.<br>For C57BL/6J mice, cells were gated on FSC-A/SSC-A to select lymphocytes, FSC-H/FSC-A to gate on single cells. SSC-A/IgM-FITC to gate on IgM- cells, then sort B220+CD43+ cells for experiment.

☒ Tick this box to confirm that a figure exemplifying the gating strategy is provided in the Supplementary Information.
